# ALOE VERA: A SHORT REVIEW

**DOI:** 10.4103/0019-5154.44785

**Published:** 2008

**Authors:** Amar Surjushe, Resham Vasani, D G Saple

**Affiliations:** *From the Department of Dermatology, Venereology and Leprosy, Grant Medical College and Sir J J Group of Hospitals, Mumbai - 400 008 Maharashtra, India*

**Keywords:** *Aloe vera*, *health and beauty*, *skin*

## Abstract

Aloe vera is a natural product that is now a day frequently used in the field of cosmetology. Though there are various indications for its use, controlled trials are needed to determine its real efficacy. The aloe vera plant, its properties, mechanism of action and clinical uses are briefly reviewed in this article.

## Introduction

The Aloe vera plant has been known and used for centuries for its health, beauty, medicinal and skin care properties. The name Aloe vera derives from the Arabic word “Alloeh” meaning “shining bitter substance,” while “vera” in Latin means “true.” 2000 years ago, the Greek scientists regarded Aloe vera as the universal panacea. The Egyptians called Aloe “the plant of immortality.” Today, the Aloe vera plant has been used for various purposes in dermatology.

### History

Aloe vera has been used for medicinal purposes in several cultures for millennia: Greece, Egypt, India, Mexico, Japan and China.^1^ Egyptian queens Nefertiti and Cleopatra used it as part of their regular beauty regimes. Alexander the Great, and Christopher Columbus used it to treat soldiers’ wounds. The first reference to Aloe vera in English was a translation by John Goodyew in A.D. 1655 of Dioscorides’ Medical treatise De Materia Medica.^2^ By the early 1800s, Aloe vera was in use as a laxative in the United States, but in the mid-1930s, a turning point occurred when it was successfully used to treat chronic and severe radiation dermatitis.^2^

### Plant

The botanical name of Aloe vera is *Aloe barbadensis miller*. It belongs to Asphodelaceae (Liliaceae) family, and is a shrubby or arborescent, perennial, xerophytic, succulent, pea- green color plant. It grows mainly in the dry regions of Africa, Asia, Europe and America. In India, it is found in Rajasthan, Andhra Pradesh, Gujarat, Maharashtra and Tamil Nadu.

### Anatomy

The plant has triangular, fleshy leaves with serrated edges, yellow tubular flowers and fruits that contain numerous seeds. Each leaf is composed of three layers: 1) An inner clear gel that contains 99% water and rest is made of glucomannans, amino acids, lipids, sterols and vitamins. 2) The middle layer of latex which is the bitter yellow sap and contains anthraquinones and glycosides. 3) The outer thick layer of 15–20 cells called as rind which has protective function and synthesizes carbohydrates and proteins. Inside the rind are vascular bundles responsible for transportation of substances such as water (xylem) and starch (phloem).^3^

**Active components with its properties:** Aloe vera contains 75 potentially active constituents: vitamins, enzymes, minerals, sugars, lignin, saponins, salicylic acids and amino acids.^4^–^6^

1. ***Vitamins*:** It contains vitamins A (beta-carotene), C and E, which are antioxidants. It also contains vitamin B12, folic acid, and choline. Antioxidant neutralizes free radicals.
2. ***Enzymes***: It contains 8 enzymes: aliiase, alkaline phosphatase, amylase, bradykinase, carboxypeptidase, catalase, cellulase, lipase, and peroxidase. Bradykinase helps to reduce excessive inflammation when applied to the skin topically, while others help in the breakdown of sugars and fats.
3. ***Minerals:*** It provides calcium, chromium, copper, selenium, magnesium, manganese, potassium, sodium and zinc. They are essential for the proper functioning of various enzyme systems in different metabolic pathways and few are antioxidants.
4. ***Sugars:*** It provides monosaccharides (glucose and fructose) and polysaccharides: (glucomannans/polymannose). These are derived from the mucilage layer of the plant and are known as mucopolysaccharides. The most prominent monosaccharide is mannose-6-phosphate, and the most common polysaccharides are called glucomannans [beta-(1,4)-acetylated mannan]. Acemannan, a prominent glucomannan has also been found. Recently, a glycoprotein with antiallergic properties, called alprogen and novel anti-inflammatory compound, C-glucosyl chromone, has been isolated from Aloe vera gel.^7^,^8^
5. ***Anthraquinones:*** It provides 12 anthraquinones, which are phenolic compounds traditionally known as laxatives. Aloin and emodin act as analgesics, antibacterials and antivirals.
6. ***Fatty acids:*** It provides 4 plant steroids; cholesterol, campesterol, β-sisosterol and lupeol. All these have anti-inflammatory action and lupeol also possesses antiseptic and analgesic properties.
7. ***Hormones:*** Auxins and gibberellins that help in wound healing and have anti-inflammatory action.
8. ***Others:*** It provides 20 of the 22 human required *amino acids* and 7 of the 8 essential amino acids. It also contains salicylic acid that possesses anti-inflammatory and antibacterial properties. Lignin, an inert substance, when included in topical preparations, enhances penetrative effect of the other ingredients into the skin. Saponins that are the soapy substances form about 3% of the gel and have cleansing and antiseptic properties.

### Mechanism of actions

1. ***Healing properties*:** Glucomannan, a mannose-rich polysaccharide, and gibberellin, a growth hormone, interacts with growth factor receptors on the fibroblast, thereby stimulating its activity and proliferation, which in turn significantly increases collagen synthesis after topical and oral Aloe vera.^9^ Aloe gel not only increased collagen content of the wound but also changed collagen composition (more type III) and increased the degree of collagen cross linking. Due to this, it accelerated wound contraction and increased the breaking strength of resulting scar tissue.^10^ An increased synthesis of hyaluronic acid and dermatan sulfate in the granulation tissue of a healing wound following oral or topical treatment has been reported.^11^
2. ***Effects on skin exposure to UV and gamma radiation:*** Aloe vera gel has been reported to have a protective effect against radiation damage to the skin.^12^,^13^ Exact role is not known, but following the administration of aloe vera gel, an antioxidant protein, metallothionein, is generated in the skin, which scavenges hydroxyl radicals and prevents suppression of superoxide dismutase and glutathione peroxidase in the skin. It reduces the production and release of skin keratinocyte-derived immunosuppressive cytokines such as interleukin-10 (IL-10) and hence prevents UV-induced suppression of delayed type hypersensitivity.^14^
3. ***Anti-inflammatory action:*** Aloe vera inhibits the cyclooxygenase pathway and reduces prostaglandin E2 production from arachidonic acid. Recently, the novel anti-inflammatory compound called C-glucosyl chromone was isolated from gel extracts.^8^
4. ***Effects on the immune system:*** Alprogen inhibit calcium influx into mast cells, thereby inhibiting the antigen-antibody-mediated release of histamine and leukotriene from mast cells.^7^ In a study on mice that had previously been implanted with murine sarcoma cells, acemannan stimulates the synthesis and release of interleukin-1 (IL-1) and tumor necrosis factor from macrophages in mice, which in turn initiated an immune attack that resulted in necrosis and regression of the cancerous cells.^15^ Several low-molecular-weight compounds are also capable of inhibiting the release of reactive oxygen free radicals from activated human neutrophils.^16^
5. ***Laxative effects:*** Anthraquinones present in latex are a potent laxative. It increases intestinal water content, stimulates mucus secretion and increases intestinal peristalsis.^17^
6. ***Antiviral and antitumor activity:*** These actions may be due to indirect or direct effects. Indirect effect is due to stimulation of the immune system and direct effect is due to anthraquinones. The anthraquinone aloin inactivates various enveloped viruses such as herpes simplex, varicella zoster and influenza.^18^ In recent studies, a polysaccharide fraction has shown to inhibit the binding of benzopyrene to primary rat hepatocytes, thereby preventing the formation of potentially cancer-initiating benzopyrene-DNA adducts. An induction of glutathione S-transferase and an inhibition of the tumor-promoting effects of phorbol myristic acetate has also been reported which suggest a possible benefit of using aloe gel in cancer chemoprevention.^19^,^20^
7. ***Moisturizing and anti-aging effect:*** Mucopolysaccharides help in binding moisture into the skin. Aloe stimulates fibroblast which produces the collagen and elastin fibers making the skin more elastic and less wrinkled. It also has cohesive effects on the superficial flaking epidermal cells by sticking them together, which softens the skin. The amino acids also soften hardened skin cells and zinc acts as an astringent to tighten pores. Its moisturizing effects has also been studied in treatment of dry skin associated with occupational exposure where aloe vera gel gloves improved the skin integrity, decreases appearance of fine wrinkle and decreases erythema.^21^ It also has anti-acne effect.
8. ***Antiseptic effect:*** Aloe vera contains 6 antiseptic agents: Lupeol, salicylic acid, urea nitrogen, cinnamonic acid, phenols and sulfur. They all have inhibitory action on fungi, bacteria and viruses.

**Clinical uses:** The clinical use of aloe vera is supported mostly by anecdotal data. Though most of these uses are interesting, controlled trials are essential to determine its effectiveness in all the following diseases.^22^,^23^

**A. Uses based on scientific evidence:** These uses have been tested in humans or animals. Safety and effectiveness have not always been proven.

**Conditions:** Seborrheic dermatitis,^24^ psoriasis vulgaris,^25^,^26^ genital herpes,^27^,^28^ skin burns,^5^,^29^ diabetes (type 2),^30^ HIV infection,^31^ cancer prevention,^32^,^33^ ulcerative colitis^34^ wound healing (results of aloe on wound healing are mixed with some studies reporting positive results^35^ and others showing no benefit^36^ or potential worsening^37^,^38^ ), pressure ulcers,^36^ mucositis,^39^ radiation dermatitis,^40^ acne vulgaris,^41^ lichen planus,^42^ frostbite,^43^ aphthous stomatitis,^44^ and constipation.^17^

**B. Uses based on tradition or theory:** The below uses are based on tradition or scientific theories. They often have not been thoroughly tested in humans, and safety and effectiveness have not always been proven.

**Conditions:** Alopecia, bacterial and fungal skin infections, chronic leg wounds, parasitic infections, systemic lupus erythematosus, arthritis and tic douloureux.

### Side effects

***Topical:*** It may cause redness, burning, stinging sensation and rarely generalized dermatitis in sensitive individuals. Allergic reactions are mostly due to anthraquinones, such as aloin and barbaloin. It is best to apply it to a small area first to test for possible allergic reaction.

***Oral:*** Abdominal cramps, diarrhea, red urine, hepatitis, dependency or worsening of constipation. Prolonged use has been reported to increase the risk of colorectal cancer. Laxative effect may cause electrolyte imbalances (low potassium levels).

**Contraindication:** Contraindicated in cases of known allergy to plants in the Liliaceae family.

**Pregnancy and breastfeeding:** Oral aloe is not recommended during pregnancy due to theoretical stimulation of uterine contractions, and in breastfeeding mothers, it may sometime causes gastrointestinal distress in the nursing infant.

**Interactions:** Application of aloe to skin may increase the absorption of steroid creams such as hydrocortisone. It reduces the effectiveness and may increases the adverse effects of digoxin and digitoxin, due to its potassium lowering effect. Combined use of Aloe vera and furosemide may increase the risk of potassium depletion. It decreases the blood sugar levels and thus may interact with oral hypoglycemic drugs and insulin.

Thus, though Aloe vera has wide spectrum of the properties and uses, some of them could be myths and some of them could be real magic. In future, controlled studies are required to prove the effectiveness of Aloe vera under various conditions.
